# Modeling trust and its dynamics from physiological signals and embedded measures for operational human-autonomy teaming

**DOI:** 10.3389/frobt.2025.1624777

**Published:** 2025-10-10

**Authors:** Abigail Rindfuss, Sarah Leary, Prachi Dutta, Ryan Chen, Torin K. Clark, Zhaodan Kong, Allison P. A. Hayman

**Affiliations:** 1 Bioastronautics Laboratory, Ann & H.J. Smead Department of Aerospace Engineering Sciences, University of Colorado, Boulder, CO, United States; 2 Cyber-Human-Physical Systems Lab, Department of Mechanical and Aerospace Engineering, University of California, Davis, CA, United States

**Keywords:** autonomous system, human-on-the-loop, psychophysiology, neurophysiology, predictive modeling, cognitive state estimation

## Abstract

Human-autonomy teaming is an increasingly integral component of operational environments, including crewed and remotely operated space missions, military settings, and public safety. The performance of such teams relies on proper trust in the autonomous system, thus creating an urgent need to capture the dynamic nature of trust and devise objective, non-disruptive means of precisely modeling trust. This paper describes the use of bio-signals and embedded measures to create a model capable of inferring and predicting trust. Data (2304 observations) was collected via human subject testing (n = 12, 7M/5F) during which participants interacted with a simulated autonomous system in an operationally relevant, human-on-the-loop, remote monitoring task and reported their subjective trust via visual analog scales. Electrocardiogram, respiration, electrodermal activity, electroencephalogram, functional near-infrared spectroscopy, eye-tracking, and button click data were collected during each trial. Operator background information were collected prior to the experiment. Features were extracted and algorithmically down-selected, then ordinary least squares regression was used to fit the model, and predictive capabilities were assessed on unseen trials. Model predictions achieved a high level of accuracy with a Q^2^ of 0.64 and captured rapid changes in trust during an operationally relevant human-autonomy teaming task. The model advances the field of non-disruptive means of inferring trust by incorporating a broad suite of physiological signals into a model that is predictive, while many current models are purely descriptive. Future work should assess model performance on unseen participants.

## Introduction

Human-autonomy teaming (HAT) is growing in relevance to operational environments, especially those where a remotely located human operator is supervising an autonomous system. Examples of these environments are remotely guided space missions, military settings, public safety, or search and rescue operations (Harel et al., 2020; J. Kintz et al., 2023). Such situations are known as human-on-the-loop (HOTL) scenarios because operators can intervene and make decisions during mission-critical events but can forfeit control to the autonomous system and take a passive role in nominal cases. This contrasts human-*in*-the-loop scenarios, where a co-located operator actively collaborates with the autonomous system for all decision-making processes. In HOTL scenarios, the operator may be remotely located from the autonomous system, such that there is spatial and possibly temporal separation. The operator thus has limited information and context, necessitating reliance on the autonomous system (O’Neill et al., 2022). As spatial or temporal separation increases, the reliance on the autonomous system also increases (Frank et al., 2016; Chancey and Politowicz, 2020).

Reliance on the autonomous system is guided by an operator’s *trust* in that system. To facilitate effective HAT, operators must appropriately trust the autonomous system (de Visser et al., 2020). Lee and See define trust as “the attitude that an agent will help achieve an individual’s goals in a situation characterized by uncertainty and vulnerability” (Lee and See, 2004). Trust guides an operator’s use of autonomy, enabling them to calibrate their perceived appropriate level of reliance and manage complex systems. The mission- and safety-critical nature of high-functioning autonomous systems means that any operator distrust, overreliance, or mistrust of the autonomous system could lead to inadequate performance or mission failure. Further, HAT tasks involve rapidly changing, dynamic environments, which can result in dynamic changes to the person’s trust in response to interactions with the autonomous system (Chandrayee Basu, 2016; Tenhundfeld et al., 2022). The operator develops an evolving perception of the autonomous system’s capabilities and faults over time, but this can also fluctuate dynamically due to discrete events that may occur while they are interacting. Capturing the temporal dynamics of trust should yield more accurate trust measurements at specific points in time. This is critical for enabling effective HAT, understanding trust relationships, and facilitating real-time applications.

Though trust is often measured as a single construct, it is multi-dimensional in nature. For example, trust dimensions may include affective, cognitive, and dispositional trust (Webber, 2008; Ayoub et al., 2021). Affective and cognitive trust are both dynamic in nature, where affective trust changes due to feelings and emotions, and cognitive trust changes due to rational and logical thoughts (Lewis and Weigert, 1985). Both components must be considered as part of overall dynamic trust, as they fluctuate during interaction with the autonomous system. Previous studies have investigated the effects of explainability on affective trust and reliability on cognitive trust (Markus et al., 2021; Kyung and Kwon, no date). The “explainability” of an autonomous system can alter the participant’s affective trust by varying the language used in communication with the participant (e.g., the level verbosity with which the autonomous system describes its confidence in its decisions). The “reliability” of an autonomous system can alter the participant’s cognitive trust by varying how often the autonomous system is correct. Both cognitive and affective trust have been extensively studied in the fields of social psychology (Yang et al., 2009), organizational leadership (Erdem and Ozen, 2003), and consumer behavior (Chang et al., 2016; Ha et al., 2016; Punyatoya, 2019), yet their applications to HAT are less understood. These dynamic facets of trust will have a large impact on human-autonomy teams, the same way that has been shown for human-human teams (Johnson and Grayson, 2005; Webber, 2008). Unlike cognitive and affective trust, the third relevant component of trust, dispositional trust, is not affected by qualities of a specific autonomous system. Dispositional trust is a person’s inherent attitude toward trusting autonomy and is considered part of their personality (Merritt et al., 2013; Ayoub et al., 2021). On the time scale of most HAT tasks, dispositional trust is a static measure, formed in the context of organizations and cultures (Webber, 2008; Ayoub et al., 2021). It may be captured through pre-experimental surveys. Dispositional trust is one primary contributor to inter-individual differences when measuring trust. It is a function of a person’s identity and demographics, and it impacts human relationships with autonomous systems (Lee and See, 2004; Chung and Yang, 2018). HAT tasks in the literature do not contain components that aim to alter both affective and cognitive trust as separate facets of trust. This suggests that current research lacks a nuanced ability to model trust and its multiple facets. Being able to model trust in a manner that is sensitive to changes in multiple forms of trust is crucial for evaluating and comprehending human-autonomy relationships.

To understand trust in HAT applications, trust must be measured accurately. Trust is historically measured through empirically determined surveys, which are considered the “gold standard” due to their validity and heritage. While these provide accurate subjective “ground truth” trust values self-reported by the participant, they are often obtrusive and static (Monfort et al., 2018; Kohn et al., 2021). Surveys are disruptive, in that they require the attentional resources of the operator or necessitate a pause in operation, rendering them unsuitable for real-world mission environments. Furthermore, surveys only capture a static measure of trust because they are often administered after multiple minute-long trials. This makes surveys insensitive to rapid, dynamic changes in trust. Thus, there is a need to develop non-disruptive, unobtrusive and continuous trust measurement techniques to ensure operational feasibility and sensitivity to trust dynamics.

There has been increasing interest in estimating trust and trust dynamics based on physiological data, such as skin conductance, electrical activity of the heart, respiration, oxygenation in the brain, electrical activity of the brain, and eye movements (Kohn et al., 2021). Critically, physiological sensors allow for unobtrusive and continuous data collection and have been shown to be promising indicators of trust without necessitating survey administration (Bandara et al., 2016; 2018). Trust is an emergent state of the brain which can be estimated by measuring activity in the central nervous system (CNS) and peripheral nervous systems (PNS). The activity of the CNS is measured by neurophysiological sensors, which focus on the brain, and the activity of the PNS is measured by psychophysiological sensors, which focus on the heart, eyes, lungs, and skin (Ajenaghughrure et al., 2020). The large body of research on these physiological correlates can be applied to predictive trust models in a HAT-specific context (Bellucci et al., 2017; Berberian et al., 2017; Krueger and Meyer-Lindenberg, 2019; Henschel et al., 2020). This research, though, has focused on human-in-the-loop HAT, while the trust dynamics of HOTL environments have not yet been modeled with physiological signals.

Embedded measures are another method of unobtrusively estimating trust and trust dynamics. These are observable behaviors captured through gaze data (e.g., where the operator is focusing their visual attention) or button-clicks (e.g., how many times the operator disagrees with the AS). These behavioral actions, inactions, or timing of actions a person takes when interacting with autonomy may be indicative of their trust levels. However, embedded measures are often influenced by additional factors, including operator workload, fatigue, and situation awareness (Parasuraman et al., 2008; Chancey et al., 2017; Xie et al., 2019). Thus, embedded measures may require additional context and can be supplementary predictors when combined with other information, such as physiological data. Previous research supports the use of embedded measures to inform trust, but they are captured after multiple minutes, so they are static measures (Hergeth et al., 2016; Kunze et al., 2019; Petersen et al., 2019; Walker et al., 2019). Embedded measures captured on the same time scale as physiological data can maximize their utility in trust estimation (Kintz et al., 2023).

Research in the literature contain various combinations of psychophysiological streams, neurophysiological streams, operator background information, and embedded measures as predictors of trust (Khalid et al., 2016; de Visser et al., 2018; Ajenaghughrure et al., 2020). While these models show promise, there is no clear justification for the combinations of sensors and measures chosen, and none have used each of these measures together in one model. Further, prior experiments that implement physiological monitoring to gauge trust have largely been single-visit lab experiments (Akash et al., 2018; Ajenaghughrure et al., 2020), making it difficult to generalize the results to different systems or HAT scenarios. Thus, there is a need to investigate trust over multiple testing sessions.

Equally important as the suite of methods used to gather data is the HAT task during which the data is gathered. An additional gap in current research is that many efforts fail to capture the complexity of HAT, particularly in HOTL tasks, when characterizing trust. Models are built on HAT tasks that are not operationally relevant or applicable to real-world scenarios (Akash et al., 2018). For example, HAT tasks that require the participant to blindly trust the autonomous system without the opportunity to make judgement calls on additional available data are less applicable to real-world scenarios (Akash et al., 2018). Some tasks involve continuous interaction with the autonomous system, characteristic of human-in-the-loop scenarios (Nikolaidis et al., 2017), and some provide simplified trusting options (Hirshfield et al., 2011; Oh et al., 2020). Other existing tasks place the participant in the same environment as the autonomous system (Hergeth et al., 2016; Morris et al., 2017), leaving a gap in our understanding for remote operational scenarios. Here, we aim to develop trust models based on HAT tasks containing features that simulate both remote operations and rich trusting options with additional available data to help increase applicability to HOTL environments and improve trust prediction in such scenarios.

In summary, there are at least four gaps concerning models of trust that need to be addressed. The first being: there is insufficient research into models built on data collected during a HOTL HAT task, limiting the applicability of current models to remote operational environments. The second gap, also related to the HAT task, is the lack of purposeful alteration of multiple dimensions of dynamic trust during the task, meaning existing models may not be sensitive to multiple dimensions affecting trust. Thirdly, current models of trust do not utilize a comprehensive suite of physiological data sensors, rather, the use of multiple sensors at once is limited. Finally, the dynamics of trust have not yet been modeled on a time scale that allows for predicting rapid changes, making current models insensitive to such dynamics.

To address the described gaps, we modeled participants’ reported trust using their dynamic physiological responses and embedded measures gathered from an operationally relevant HOTL HAT task. The HAT task alters multiple dimensions of trust to capture a comprehensive measure of overall trust in the model. We aim to improve upon previous trust models by leveraging a wide physiological suite of measures for use as predictors. This research develops metrics and models capable of capturing inherent trust dynamics that are transferable across a wide range of tasks.

## Methods

### Task

**FIGURE 1:**
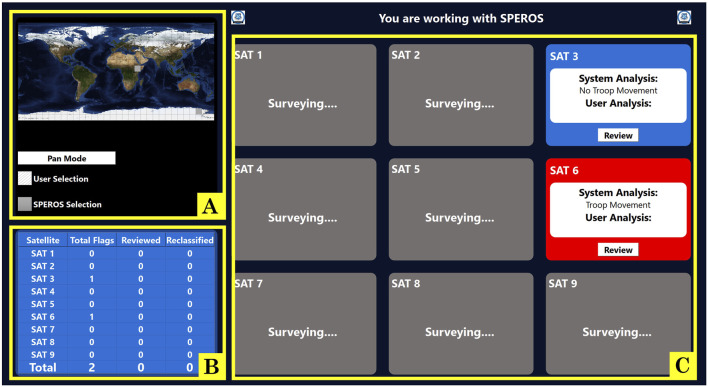
Home screen, where users access the map and select classifications to review. Two satellite classifications appear every 30 s, with red indicating a classification of troop movement and blue indicating a classification of no troop movement. The participant may select two areas in the map every 30 s. **(A)** scan guidance map. **(B)** satellite summary table. **(C)** satellite overview panels.

**FIGURE 2:**
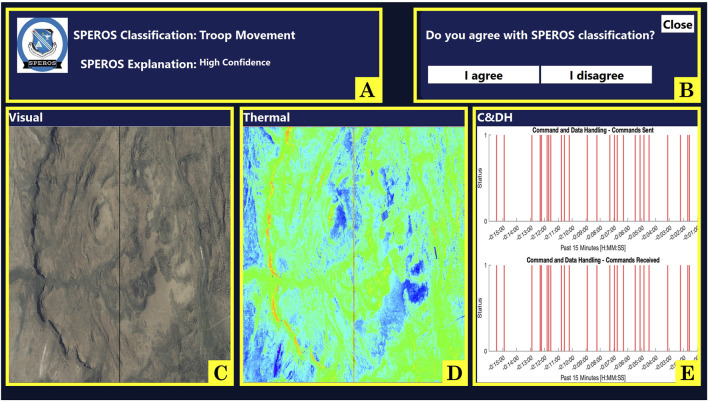
Satellite review screen, where users may assess the data and agree or disagree with the system’s recommendation. On this page, participants are provided with the satellite’s recommendation, an explanation of its confidence in its decision (which is meant to alter affective trust), a visual image, a thermal image, a command and data handling image (where the combination of information from the three images is meant to manipulate cognitive trust if subjects identify a conflict between the information and the recommendation of the autonomous system), and agree or disagree buttons. **(A)** autonomous system recommendation and confidence explanation. **(B)** “I agree” and “I disagree” buttons. **(C)** satellite visual image. **(D)** satellite thermal image. **(E)** command and data handling (C&DH) information.

To study trust dynamics, we developed an operationally relevant task where participants teamed with a simulated autonomous system with the goal of identifying ground troop movement in satellite data. Complete details of the task design are discussed previously (Sung et al., 2024) and summarized here. During the task, the participant interacted with the simulated autonomous system through a computer screen. The autonomous system received information from heritage satellites. The system classified satellite data as containing troops or not containing troops, analyzing simulated data from up to nine satellites. It then conveyed the classification for a given satellite to the participant on their computer screen, as seen in Figure 1. The participant had the option of “reviewing” the classification of the autonomous system, though this was not required. If choosing to “review,” the autonomous system provided the participant with three pieces of satellite data and a statement expressing how confident the autonomous system was in its classification of the data (Figure 2). To alter the cognitive dimension of trust, the autonomous system was fallible such that its classification was not always correct. The autonomous system had a “low reliability” condition in which the system is correct in its classification 67% of the time and a “high reliability” condition in which the system was correct 84% of the time. The participants were not informed of the reliability percentages, nor were they aware of the reliability setting during each session. Rather, subjects were told they would be working with a different autonomous system in each session. To alter the affective dimension of trust, the autonomous system would use either terse, robotic-like language (in the “low explainability” condition) or use naturalistic language (in the “high explainability” condition). Like reliability, participants were also not informed of the explainability conditions during testing. Varying explainability and reliability resulted in four different autonomous systems, each possessing a unique combination of reliability and explainability. An additional method implemented to alter overall trust was confidence matching, where the level of reliability and the level of the system’s communicated confidence either aligned or were mismatched (Sung et al., 2024). To increase co-reliance, the participants were asked to also assist the autonomous system in tasking satellite image acquisition. In this objective, the participant interacted with a map (Figure 1A) to suggest places on the globe for satellites to image. This “scan guidance” task required the participant to strategically allocate their attentional resources. The division of attention between the two tasks was designed to provide insight into the participants’ trust in the autonomous system teammates, depending on the amount of time they dedicated to monitoring the autonomous system or selecting locations on the map.

### Experimental protocol

The experiment consisted of one training session and four testing sessions. Participants interacted with a unique autonomous system during each testing session. Each session was conducted on different days with no more than 1 month between the training session and the fourth testing session. Twelve participants (5 F and 7 M, ages 25.6 +- 8.26 years) completed this study. The participant pool included people with diverse educational backgrounds: one individual held a Ph.D., three had Master’s degrees, and eight either held or were pursuing Bachelor’s degrees. Their fields of study included astrophysics, aerospace engineering, mechanical engineering, geography, biotechnology, and computer science. The experimental protocol was approved by the University of Colorado Boulder’s Institutional Review Board (IRB) under protocol number 23-0103.

The training session began with operator background surveys. These were selected based on a prior study identifying significant associations between trust dynamics and personal characteristics (Chung and Yang, 2024). Participants filled out the “Extraversion” and “Agreeableness” sections of the Big Five Factors of Personality (Donnellan et al., 2006) survey, the “High Expectations” component of the Perfect Automation Schema (PAS) (Merritt et al., 2015), the “Masculinity” dimension of the Cultural Values Scale (CVS) (Yoo et al., 2011), the Propensity to Trust (PT) (Merritt et al., 2013) survey, the Automation Induced Complacency Potential (AICP) Scale (Merritt et al., 2019), and the “Performance Expectancy” as well as “Effort Expectancy” sections of the Unified Theory of Acceptance and Use of Technology (UTAUT) survey (Venkatesh et al., 2003). Subdimensions of these scales were administered, as recommended by Chung and Yang (Chung and Yang, 2024), *in lieu* of the full surveys to prevent survey fatigue. Participants also provided demographic and lifestyle information, including age, sex, race, ethnicity, dominant hand, experience with video games, experience with robotic systems, navigational aid use, experience with aerospace relevant displays, and experience with ground troop or other military monitoring systems.

Next, participants were trained on the task with a slideshow. They were instructed to click through it at their own pace and encouraged to ask questions as needed. The training slides presented relevant information about the task objectives and background. They also provided a detailed breakdown of the task screens, buttons, and data, including example images and trust slider pop-up instructions. Subjects were shown how the information on the review panel may agree or disagree with the autonomous system recommendation. They are also told that the autonomous system has access to additional information provided by the satellites to help it make its determination, which they as the operator do not have access to. Thus, we do not explicitly tell them it is fallible, but leave it ambiguous as to whether or not they should trust the system. The slides emphasized that participants should rate their trust based on how the autonomous system performed on the current task, ignoring how they thought it would perform on any other task. Participants were given the Lee and See definition of trust (Lee and See, 2004) and were directed to use this definition when completing the trust sliders. The slides also informed participants of the compensation structure of combined base pay and performance bonuses. At the end of the training slideshow, they completed an oral quiz to ensure they understood all facets of the task.

As the final part of the training session, participants performed three hands-on practice trials with a version of the simulated autonomous system that was 100% correct and always explained that it was 100% confident in its assessment (subjects were told that this system is always correct so as not to bias their understanding of the task). The goal of the hands-on training is to get the participant accustomed to the functionality of the task and to practice finding “troop movement” in data provided by the autonomous system. Physiological data and embedded measure data were not collected during the practice trials. After the training session, participants were scheduled for the subsequent four testing sessions, each with a separate visit to the lab.

**FIGURE 3:**
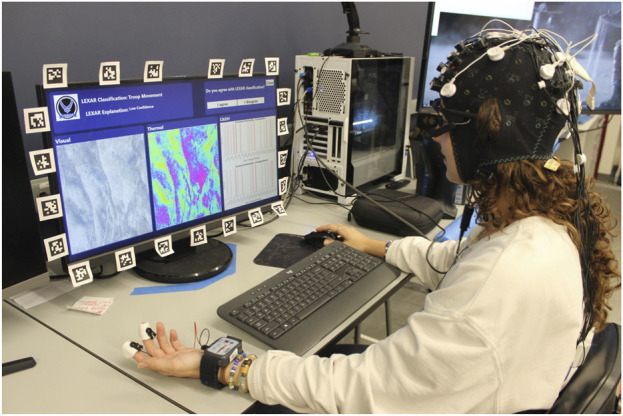
A person wearing the sensors while doing the HAT task.

**FIGURE 4:**
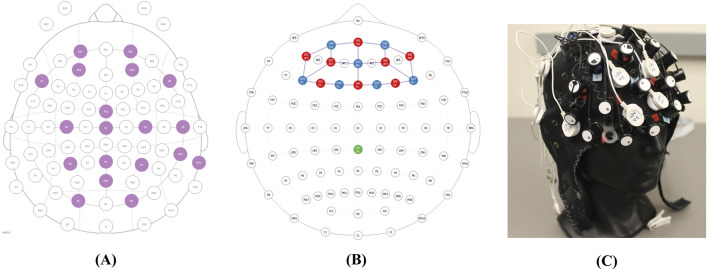
Integrated neurophysiological montages. **(A)** EEG Montage. **(B)** fNIRS montage. **(C)** Montages combined in the head cap.

When participants arrived for the testing session, they first filled out a brief questionnaire where they indicated the amount of sleep they obtained the previous night and confirmed that they had not consumed alcohol within 6 hours prior to the session. Then, participants completed a psychomotor vigilance test on the computer screen to gauge their motoric alertness and sustained attention (‘Psychomotor Vigilance Task’, no date). Next, two trained personnel assisted in placing sensors on the participant (Figure 3). The suite of sensors includes a BIOPAC Binomadix 3-lead electrocardiogram (ECG) montage, a BIOPAC Binomadix respiratory (RSP) chest band, two BIOPAC Binomadix electrodermal activity (EDA) electrodes on the pointer and middle fingers, a Neuroelectrics Enobio 19-lead electroencephalogram (EEG) montage, a 15-optode (8 sources, seven detectors, 20 source-detector pairs) NIR-X Sport Functional Near-Infrared Spectroscopy (fNIRS) montage (Figure 4B), and Pupil Lab’s Pupil Core eye-tracking glasses. The monopolar EEG montage (Figure 4A) contained the temporal lobe, occipital lobe, parietal lobe, central lobe, and pre-frontal cortex. Both the EEG montage and the fNIRS montage were integrated in the same head cap (Figure 4C).

After all sensors were donned and calibrated, a short script was read to the participants to briefly remind them of the objectives of the task and how we would collect measures of trust. They were reminded of the Lee and See definition of trust to be used during the experiment. It was also emphasized that the autonomous system they were working with on that day was different from those of prior days. Prior to the start of the first trial, the participants completed a 2-min-long pre-experimental baseline, where they were instructed to stare at a crosshair on the computer screen, but not to think about the task. Once the pre-experimental baseline ended, the first trial immediately began. For trials 2-6, the pre-trial baseline period was 45 s.

Each trial was split into eight 45-s epochs (i.e., subdivisions of time over which physiological and embedded measures are captured). To capture trust, the task paused at the end of each epoch, and a screen appeared that asked the participant to rate their agreement with the statement “I trust this autonomous system” on a continuous scale from “Not at all” to “Completely”. This analog visual slider method was minimally disruptive, as it briefly captured a single trust value and allowed participants to quickly return to the task. After rating their trust, the task continued. This process was repeated for the entirety of the 6-min trial, which yielded eight trust slider reports per trial to capture trust dynamics during the trial. After the trial, we also administered the Jian Trust in Autonomous Systems survey (Jian et al., 2000), as a gold-standard, but static measure of trust. Lab Streaming Layer (LSL) time-synchronized the task and six bio-signal sensor streams and dynamic trust measures.

**FIGURE 5:**
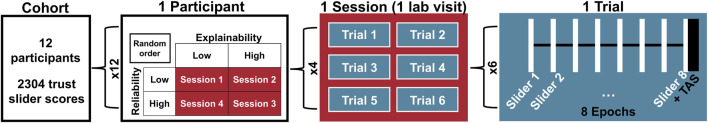
Visual representation of the experimental protocol and data collection.

Over one testing session, a participant performed six trials total; over the experiment, a participant completed four testing sessions on four different days. They collaborated with a different autonomous system for each of the four sessions, each with a different combination of reliability and explainability. The testing protocol is outlined in Figure 5. Across all four testing sessions per each of the 12 participants, the experiment yielded 2,304 epochs total, which corresponds to 2,304 trust slider reports.

### Data cleaning and feature extraction

The raw data from each sensor stream was imported into MATLAB (version 2024a), cleaned, and separated into 45-s epochs prior to feature extraction. Physiological and embedded features were calculated during each 45-s epoch between trust slider reports. Data cleaning and feature extraction for ECG, EDA, and respiration data was done identically to the methods described in Richardson et al. (Richardson et al., 2025). In summary, The ECG data was filtered using a highpass filter with a passband frequency of 1 Hz to remove baseline drift, a lowpass filter with a passband frequency of 100 Hz to remove electromyographic noise, and an infinite impulse response (IIR) Butterworth bandstop filter with a lower cutoff frequency of 59 Hz and an upper cutoff frequency 61 Hz to remove powerline interference. R-DECO was used to extract R-peaks from the cleaned ECG signal, all of which were subsequently visually inspected and confirmed. For EDA, to identify motion artifacts, values outside of 1–40 µS were identified and removed. Next, a Savitzky-Golay finite impulse response smoothing filter of polynomial order three was used to smooth the EDA signal. Ledalab’s continuous decomposition analysis was used to decompose the EDA signal (downsampled to 10 Hz, as recommended) into tonic and phasic components. The toolkit returned the deconvolved components and skin conductance response (SCR) characteristics. Respiration data was filtered using an IIR Butterworth band-stop filter with a lower cutoff frequency of 0.05 Hz and an upper cutoff frequency of 3 Hz to remove baseline drift and high frequency noise while preserving breathing rates between 3 and 180 breaths per minute. Zero-phase digital filtering was used to eliminate the non-linear phase distortion of IIR filtering for both ECG and respiration. ECG yielded 28 features, EDA yielded 63 features, and respiration yielded 28 features. Surveys related to operator background were administered during the training session only. The features from the physiological data, embedded measures, and operator background information resulted in a total of 680 features, which are detailed below. No features were calculated from interactions.

fNIRS data was analyzed as follows: The data acquisition software for the fNIRS system, Aurora (version 2021.4), automatically applies the Modified Beer-Lambert law to transform raw voltage data into oxygenated hemoglobin (HbO) and deoxygenated hemoglobin (HbR) concentrations. HbO and HbR were bandpass filtered with a lower cutoff frequency of 0.016 Hz and an upper cutoff frequency of 0.5 Hz to remove linear drift and cardiac artifacts (NIRx Medical Technologies, 2020). The following features were extracted for each channel: maximum amplitude (HbO only), minimum amplitude (HbR only), time to maximum amplitude (HbO only), time to minimum amplitude (HbR only) mean, variance, skew, kurtosis, RMS, slope, and area under the curve. In total, 360 fNIRS features were created.

The EEG data was analyzed as follows: A fourth order Butterworth bandpass filter was used with a lower cutoff frequency of 0.5 Hz and an upper cutoff frequency of 55 Hz. The EEGLAB MATLAB toolbox (version 2024.0) (Delorme and Makeig, 2004) was then used to conduct independent component analysis (ICA). Components were manually rejected if they were indicative of eye artifacts, such as blinks or saccades. The data was then reconstructed from the remaining independent components. The power spectral density (PSD) of the delta (1–4 Hz), theta (4–8 Hz), alpha (8–13 Hz), beta (13–30 Hz), and gamma (30–80 Hz) bands was calculated using standard tools in EEGLAB. This was done for each of the 19 EEG channels. The first features extracted were the power spectral densities in each band for each channel, thus yielding 95 features. Then, the power spectral density of channels centered on brain regions of interest were averaged to generate region-specific features. These regions included the 1) pre-frontal cortex, 2) central cortex, 3) parietal lobe, 4) occipital lobe, 5) temporal lobe, and 6) entire head. This yielded an additional 30 features. All individual channel features and region features were used, totaling 125 features.

The Pupil Labs’ Pupil Core (version 3.5.1) (Kassner et al., 2014) headset measured pupil diameter, blink duration, blink rate, saccades between pre-defined areas of interest on the screen, and fixation durations on areas of interest. Each were retained as features. There were 21 features associated with pupils and blinks and 16 associated with fixations and saccades, yielding 37 total eye features.

Finally, 16 button-click-based embedded measures were collected. A full list of embedded measures is provided in Table 1.

**TABLE 1:** List of task-specific embedded measures.

Embedded measures
1. Session number (1-4)
2. Trial number (1-6, per session)
3. Epoch number (1-8, per trial)
4. Total time spent on the review screen per epoch
5. Average time on the review screen per epoch
6. Percent of satellites reviewed per epoch
7. Percent of satellites ignored per epoch
8. Percent of agreement with the autonomous system per epoch
9. Percent passive agreement with the autonomous system per epoch
10. Average time between clicking “review” and classifying the satellite per epoch
11. Percent of satellites re-reviewed per epoch
12. Number of map area selections made per epoch
13. Percent of allowable map interactions made per epoch
14. Post-trial analog slider: “How much did you rely on the system’s recommendation vs. additional data?”
15. Number of screen switches per epoch
16. Autonomous system explainability mode (per session)

There were multiple epochs that contained missing data for entire trial(s) or individual epoch(s) from various sensor streams. Data was imputed by filling in missing data with the average of the rest of the trial if an epoch was missing, the average of the rest of the session if a trial was missing, and the average of the remaining three sessions if a data stream was absent for an entire session. An entire session’s worth of missing data only occurred twice. In total, 0.24% of the data was imputed. To assess the impact of our chosen imputation method, we also implemented a feed-forward imputation method (i.e., using the most recent available data as a proxy for missing data) and found very similar predictive accuracy, suggesting our imputation method was not critical for how models were built.

### Feature versions

For all physiological features, the baseline values were removed to account for inter-individual differences among participants. There exist multiple methods of baselining physiological features (e.g., comparing a raw response to a participant’s pre-trial resting response). Since our model contains many different types of features ranging from neurophysiological to psychophysiological, the ideal method of baselining could be different for each. Multiple methods, or “versions,” were calculated to be potential predictors for the model. Feature versions are adapted from Richardson et al. (2025). Raw features were calculated over the 2-min pre-experimental baseline, 45-s pre-trial baseline, and active 45-s epochs, as defined in the Experimental Protocol. These raw values are used to create eight feature “versions,” which include: 1) subtracting out the participant’s per-trial pre-trial baseline from that trial’s active period values, 2) subtracting out the participant’s mean pre-trial baseline from their active period values, 3) subtracting out the participant’s pre-experimental from their active period values, 4) dividing the active period values by that participant’s per-trial pre-trial baseline, 5) dividing the active period values by that participant’s mean pre-trial baseline 6) dividing the active period values by that participant’s pre-experimental baseline, 7) standardizing the active period values by subtracting that participant’s mean and dividing by their standard deviation of those values, and 8) mean-centering the active period values by subtracting that participant’s mean of those values. All eight versions were applied to psychophysiological features, including ECG, EDA, respiration, and eye data. For EEG and fNIRS, the approaches to baseline these signals are more standardized in their respective fields. With EEG features, only version 4 (dividing by the per-trial pre-trial baseline) was applied. For fNIRS features, only version 1 (subtracting the per-trial pre-trial baseline) was applied. Applying only one baseline technique also reduced the risk of expanding the feature space unnecessarily due to the large quantity of channels on each device.

### Model building and evaluation

#### Predictive model

**FIGURE 6:**
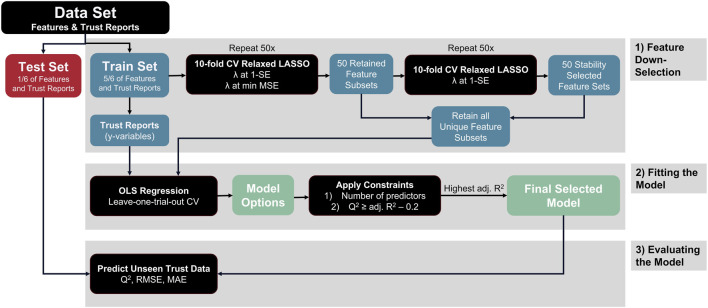
Flow chart of the feature down-selection and model-fitting process. Note that this flow chart represents one single test-training split. Our modeling process involved 10 separate test-training splits.

The predictive model on unseen trials was created by fitting the suite of psychophysiological measures, neurophysiological measures, operator background information, and embedded measures to the trust slider reports. The trust slider values were used as continuous, dynamic ground truth measurements, as these have been shown to be strongly correlated to the Jian et al. Trust in Automation Survey, using this same task (Leary et al., 2024). To assess model performance on trials not used in the model building process, we implemented external validation. External validation holds out a set portion of data for model building and the remainder is used for model evaluation. Thus, feature selection was conducted on 83% of the trials (training data) and the remaining 17% of the trials (test data) was held aside until after the final model was selected and fit. Leave-one-trial-out-per-session (LOTOPS) Monte-Carlo cross validation (MCCV) was implemented to split test and training data. Thus, for each distinct participant and session, one random trial out of six was withheld to be test data, resulting in 1/6th, or 17%, of the data being set aside. The model was fit using the process described in the next paragraph on 5/6th, or 83% of the observations. The model was then evaluated on the test dataset to evaluate the model performance and generalizability. Finally, because the original train/test split was random, the entire process was repeated 10 times, resulting in 10 unique models, known as MCCV, to provide the most realistic picture of the model building pipeline’s performance for future unseen predictions. Finally, the entire data set was used to fit the final model presented for descriptive evaluation using adjusted R^2^ as the metric of performance. The modeling process is summarized in Figure 6.

Our feature selection and modeling fitting approach builds on previous cognitive state estimation research and is useful for shrinking a large set of potential predictors (Buchner, 2022). The model was fit with ordinary least squares (OLS) multiple linear regression models (of the form *y = β*_*0*_ *+ β*_*1*_ *X*_*1*_ *+ . β*_*p*_ *X*_*p*_) using predictor variables to estimate the self-reported trust scores for each epoch (Buchner, 2022). The independent variable, trust (*y*), is estimated by dependent variables, or predictor variables (*X*_*1*_ *, X*_*2*_ *, ., X*_*p*_), each of which is multiplied by a coefficient (*β*_*0*_ *, β*_*1*_ *, … , β*_*p*_), with *β*_*0*_ being the y-intercept. Predictor variables were selected using Least Absolute Shrinkage and Selection Operator (LASSO) shrinkage. Ten-fold cross validation relaxed LASSO was used to identify two sets of predictors by 1) setting the shrinkage coefficient λ at the one standard error (1-SE) location and 2) setting λ at the minimum mean squared error (MSE) location (Tibshirani, 1996; Meinshausen, 2007). The λ value describes the amount of coefficient shrinkage occurring during down-selection, where λ = 0 implies zero shrinkage (all features are retained), and λ = ∞ implies that no features are retained. The 10-fold cross validation allows for λ values to be selected based on MSE, where the 1-SE location denotes the largest λ at which the MSE is within one standard error of the lowest MSE, and the minimum MSE location represents the minimum λ value (Hastie et al., 2009; Krstajic et al., 2014). Ten-fold cross validation was used to balance bias and variance when selecting the λ parameter (Breiman and Spector, 1992). Because each run of LASSO is a stochastic process, this process was repeated 50 times, generating 100 sets of down-selected predictors (50 at λ = 1-SE and 50 at λ = min MSE). Each unique model solution was retained. Then, a second round of feature down-selection was conducted with 50 additional runs of LASSO using only the retained features, identifying predictors by setting λ at the 1-SE location. This was done to reduce instability in the predictor sets (Meinshausen and Bühlmann, 2010). For each of these 50 models, the β coefficients and a y-intercept were fit. To determine the best of the 50 models, adjusted R^2^ was calculated. Q^2^, which is a measure of a model’s predictive ability, was also fit using exhaustive leave-one-trial-out (LOTO) cross validation. As such, an OLS using the LASSO-determined predictors was fit on 239 trials. The model was then used to predict the remaining single trial. This was done iteratively for each trial to generate a LOTO Q^2^. The selected model’s predictions were restricted to the bounds of [0,1], as trust slider reports do not exceed that range. Any prediction lower than 0 or higher than one was replaced with 0 and 1, respectively. To avoid overfitting, models were eliminated based on two constraints: 1) the number of predictor variables used in the model must not exceed 1/5th the number of observations, and 2) the LOTO Q^2^ must be within 0.2 of the adjusted R^2^ (Richardson, 2024). After constraints were applied, the model option with the highest R^2^ was chosen as the “selected” model. All feature selection was done in MATLAB 2024a.

Once the final model was selected, its performance was evaluated on the previously retained test data. A “test” Q^2^ was calculated to assess the model’s predictive capabilities on the unseen trials. Differences between model predictions and unseen trust slider reports were also calculated, in terms of root mean square error (RMSE) and median absolute error (MAE). The train/test split was repeated 10 times in MCCV. To qualitatively assess the model’s performance in predicting dynamic changes in trust, the per-epoch reported trust and predicted trust were compared side-by-side. Thus, for every 45 s of the test dataset, the trust prediction and corresponding ground truth value were plotted to observe the model’s ability to statically predict temporal fluctuations. This was done for each of the 10 MCCV train/test splits.

#### Descriptive model

In addition to creating 10 models using train/test splits to assess predictive performance, an “overall” model was also created using 100 percent of the dataset. This was done to observe the model fit (R^2^) and the features down selected by LASSO. In this process, 10-fold cross validation was repeated 50 times and constraints were applied, identical to the method described in Section 2.5. The R^2^ associated with the selected model in this process is the overall descriptive R^2^, and it is the measurement used to describe the model fit on the complete data set. This overall model was also used to extract the names of features that were retained as predictor variables in the model.

## Results

To assess the predictive fit, the adjusted R^2^ per model, the Q^2^ calculated per model (Model Q^2^), the Q^2^ used to measure the model’s predictive capabilities on the test data (Test Q^2^), the RMSE of the trust predictions in the test set, the MAE of the trust predictions in the test set, the number of predictors retained in the model, and the number of estimates that went outside the [0-1] range of the trust slider, and thus were capped (capped predictions) are summarized in Table 2. The mean and standard deviation across all 10 predictive models is shown at the bottom of the table. Note that our most important metric of predictive performance on unseen trials yielded a mean test Q^2^ of 0.64 ± 0.05. The average descriptive adjusted R^2^ across all 10 predictive models is 0.83 ± 0.006. An average of 320 ± 60 predictors were used in the model, across 10 train/test splits. The RMSE was an average of 0.13 ± 0.005, indicating that measures were generally within 13% of the scale. Across all measures, the standard deviations are low, demonstrating consistency in model performance regardless of the train/test split.

**TABLE 2:** Summary of 10 test-training splits. The adjusted *R* ^2^ describes the model’s ability to estimate trust in the training data. The “Model Q^2^” describes the Q^2^ calculated during cross validation, which was used in model selection. The “Test Q^2^” describes the model’s ability to predict unseen test data. The “Predictors” column refers to the number of predictors that resulted from the LASSO down-selection. The “Capped Predictions” are the number of observations (out of 384 test observations) that were restricted to fit the bounds of [0,1].

Split	Adj *R* ^2^	Model Q^2^	Test Q^2^	RMSE	MAE	Predictors	Capped predictions
1	0.82	0.74	0.68	0.12	0.073	263	2
2	0.83	0.75	0.67	0.12	0.076	260	5
3	0.84	0.72	0.58	0.13	0.088	419	5
4	0.83	0.66	0.66	0.13	0.083	286	8
5	0.84	0.64	0.67	0.13	0.082	387	2
6	0.84	0.74	0.55	0.13	0.085	372	12
7	0.83	0.66	0.69	0.12	0.075	234	0
8	0.83	0.74	0.63	0.12	0.079	323	2
9	0.83	0.72	0.68	0.13	0.076	321	2
10	0.83	0.75	0.61	0.13	0.080	280	1
Mean (σ)	0.832 (0.006)	0.71 (0.04)	0.64 (0.05)	0.126 (0.005)	0.080 (0.005)	320 (60)	4 (4)

**FIGURE 7:**
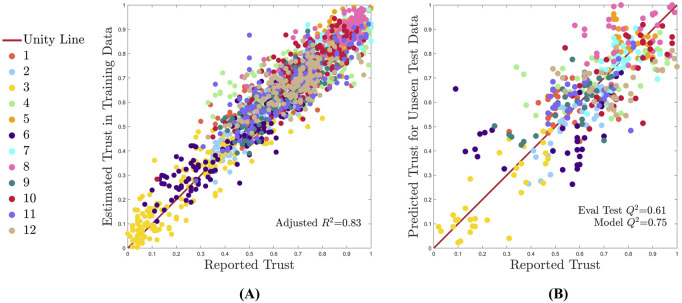
One random example MCCV training-test split (split 10), where the model is assessed for fit on the training data and for predictive accuracy on the test data. All subjects are shown by a unique color as identified n the legend, and perfect model performance is represented by the unity line. **(A)** Model descriptive fit on training data. **(B)** Model predictive fit on test data.

As a visual representation of one of the predictive model fit capabilities in a single train/test split, trust predictions are plotted against ground truth trust reports, as seen in Figure 7. The unity line provides a reference for perfectly accurate predictions. Figure 7A shows one example MCCV split with the training dataset and Figure 7B depicts the unseen test dataset (16.6% of the data). Each of the 12 participants are plotted in a different color.

**FIGURE 8:**
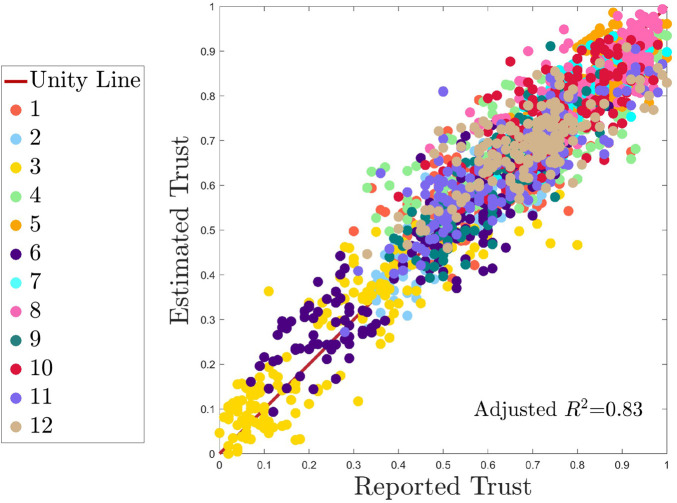
Overall descriptive fit of the model. Note that this plot was generated without any MCCV train/test splits. It is used to assess the model’s descriptive capabilities on the entire dataset. All subjects are shown by a unique color as identified n the legend, and perfect model performance is represented by the unity line.

The descriptive model fit for all data is visualized in Figure 8. This is the fit of the model built on 100% of the data, in the “overall” model. The unity line and participant colors are identical to those of Figure 7. The adjusted *R* ^2^ of the descriptive model is 0.83. The RMSE is 0.089 and the MAE is 0.064. The number of predictors in the descriptive model is 279. For each feature category or sensor type (e.g., fNIRS, EDA, embedded measures), the number of features in each category and the number of features retained per category are shown in Table 3.

**TABLE 3:** Feature categories and down-selection.

Feature category	Features created	Features retained	Percent retained
EEG	125	35	28%
fNIRS	360	157	44%
Eye	37	10	27%
ECG	28	9	32%
Respiration	28	9	32%
EDA	63	26	41%
Operator Background	25	14	56%
Embedded Measures	16	11	69%

**FIGURE 9:**
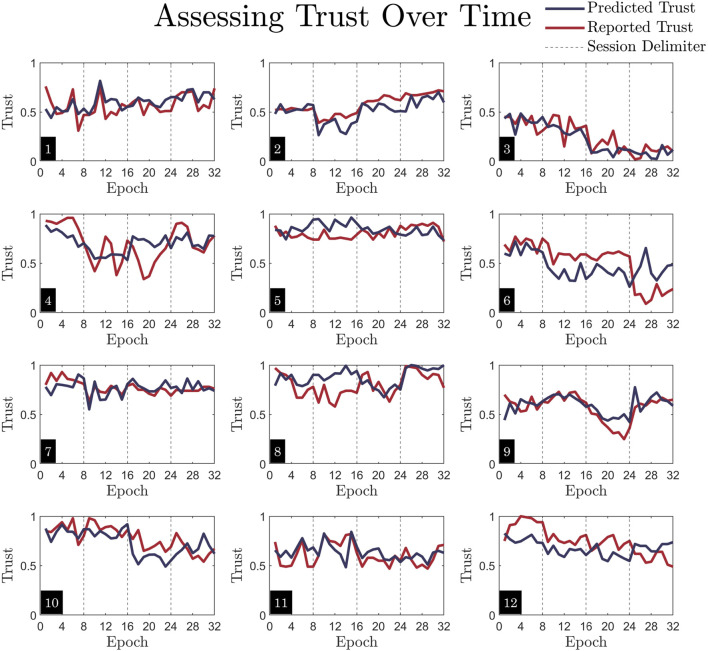
Visualization of temporal fluctuations in trust. Each subplot is one subject. These plots correspond directly to Figure 7, as they are made with the same MCCV test/train split. The data shown here is identical to that of Figure 7B, where the test dataset is used to assess predictive accuracy, only here both predicted trust and reported trust are plotted together against time. Epoch number (1-32, with eight epochs per trial) indicates each 45s time segment. Here, one trial per session makes up the test dataset. Note that the vertical dashed lines indicate separate sessions, so each of the four sections should be interpreted as unique days. While the plot lines connect across these delimiters, the data is not continuous.

The per-epoch predictive capabilities of model on unseen trials are also assessed on each participant, depicted in Figure 9. The reported, ground-truth trust is plotted in red, and the model’s predicted trust is plotted in navy. The model can predict fluctuations in trust between epochs during unseen trials for each participant, as the navy lines generally trend in the same direction as the red, ground truth trust values. While the model captures the dynamics per subject, it also captures individual differences between subjects. For example, Participants three and five are consistently low or high trusters, whereas participants such as four and six have a wider range of values across the trust scale. To quantify the model’s predictive accuracy per-participant, the RMSE for each of the 12 participants is listed in Table 4.

**TABLE 4:** Summary of RMSE per-participant. These values correspond directly with the predictions in Figure 9.

ID	1	2	3	4	5	6	7	8	9	10	11	12
RMSE	0.10	0.087	0.093	0.15	0.10	0.21	0.072	0.14	0.10	0.12	0.10	0.16

## Discussion

### Model performance

The developed modeling approach demonstrated the ability to both describe and predict subject trust. The model achieved a descriptive accuracy of 0.83 (given by R^2^) and a predictive accuracy of 0.64 (given by Q^2^), which is an improvement (Kintz et al., 2023) or is comparable with (Akash et al., 2018; Guo and Yang, 2021; Richardson et al., 2025) other models available in the literature (Rodriguez Rodriguez et al., 2023). Note that this work achieved this level of accuracy by collecting data from six physiological sensors, capturing a range of high and low trust implementing robust mathematical modeling techniques (e.g., external validation, stability selection, methods to prevent overfitting), and fitting the model to dynamic trust reports, which advances the field of research and addresses gaps present in past models.

A major focus of this effort was assessing model predictive performance and generalizability. This is important for translation into application because it represents how the model would be deployed and used in future operational settings, where the model is needed to predict performance on events happening in real time. Our modeling approach accurately predicts operator trust reported during left-out trials for each participant, for a range of high and low trust reports. The utility of the model is due to multiple factors. First, it harnesses a large suite of neurophysiological features, psychophysiological features, embedded measures, and operator background information. These measures are unobtrusive and can be collected in operational environments. Second, the model is built based on an operationally relevant HOTL HAT task that collects dynamic trust reports and purposely aims to affect multiple dimensions of trust. Thus, this research addresses gaps identified in reviews by Kohn, et al. and Ajenaghughrure et al. (Ajenaghughrure et al., 2020; Kohn et al., 2021). The inclusion of a variety of features fit to dynamic trust reports allows for high predictive capabilities when the model is assessed on left-out data. The RMSE was an average of 0.13, indicating that measures were within 13% of the scale (Table 2). With this level of granularity, being within 13% of the scale is an accomplishment and demonstrates the model’s strength in being useful in HAT scenarios outside of our task. Additionally, the low standard deviation values in Table 2 show that these predictive models are consistent across all 10 test train splits, indicating that the model is stable and reliable, even though the test/train data is split differently each time.

### Data streams

The inclusion of a multitude of physiological sensors during data collection yields a novel model that implements a variety of features as predictors of trust. For each model fit in the process, features from each physiological sensor were retained, and thus provided valuable information that enhanced the predictive capabilities of the model. In prior work, features derived from ECG, EDA, respiration, eye-tracking, EEG, and fNIRS data have each been explored, but have never been combined for use in a single model as was done in this effort. A review by Ajenaghughrure et al. found the maximum number of physiological signals combined in any study that assessed trust is three (Ajenaghughrure et al., 2020), including outside of HAT contexts but to study human-human trust as well. Eye movements, EDA, and ECG have been used together to assess trust between active and passive technology users in the context of multi-user systems, customer service, healthcare, and workflow (Xu and Montague, 2013). Blood pressure, respiration rate, and electrical emotional rating scales were used in another study that assessed interpersonal trust in the context of gratitude and relationships with strangers (Drążkowski et al., 2017). Not included in the review by Ajenaghughrure et al. is a third study that collected respiration, ECG, and pupillometry data to model trust (as well as situation awareness and mental workload) in an HAT scenario (Buchner et al., 2025). One additional study used four psychophysiological sensors (ECG, respiration, EDA, and pupillometry data), also to model trust, workload, and situation awareness in a HAT scenario (Richardson et al., 2025) These studies did not include neurophysiological data temporal resolution compared to the peripheral nervous system sensors. This is important since psychophysiological measures are non-specific, and so may be confounded by other aspects of the task, such as engagement or an action the operator might take each trial. Other studies on trust assessment have combined psychophysiological and neurophysiological sensors to monitor both the peripheral and central nervous system, respectively, however the maximum number of sensors used in these studies was two. For example, multiple experiments have combined EDA and EEG, electrooculography and EEG, or eye-tracking and functional magnetic resonance imaging (fMRI) for measuring trust (Boudreau et al., 2009; Marzi et al., 2014; Ma et al., 2015; Hu et al., 2016; Akash et al., 2018; de Visser et al., 2018; Prochazkova et al., 2018; Ajenaghughrure et al., 2020). The landscape is ever changing and our own results in prior effort have pushed the boundary on total number of sensors, up to four (Richardson et al., 2025). Our results in this study are given by a combination of six sensors (ECG, EDA, respiration, eye-tracking, fNIRS, and EEG), and suggest that 1) combining both peripheral and central nervous system measurements, and 2) implementing multiple types of both neurophysiological and psychophysiological sensors, offers a more comprehensive understanding of trust as related to physiological monitoring. Further, we accounted for the non-specific nature of psychophysiological measures in this research by ensuring the trust slider movement was not included in the data analysis and accounting for any time-based effects (e.g., relaxation, novelty, learning, or boredom) by including trial number as a feature available to the model. We also fit our models to the gold-standard Jian Trust in Automation Scale which yielded similar outcomes as the trust sliders, helping to ensure high ecological validity, Thus, we believe this work better fulfills the suggestion outlined by Kohn, et al., which highlighted the need for a better model of trust based on the measurable components of trust (Kohn et al., 2021).

The use of physiological data to model trust in HOTL scenarios is under studied. However, it has been researched in many other areas of HAT, including assisted spacecraft docking scenarios and autonomous road vehicle environments (Hergeth et al., 2016; Buchner et al., 2025). Our results add to the field by applying methods comparable to previous research to a new HOTL remote supervision scenario, as it was not previously clear that modeling trust in this way could be generalized to all forms of HAT. Our results suggest that, in addition to its previous applications, physiological data is also useful to model and predict trust in a HOTL environmental context, addressing this gap.

Personality features included in the model were: AICP, PAS, PT, UTAUT relative advantage, UTAUT perceived ease of use, and UTAUT complexity scores, calculated from pre-experimental surveys, as described in Section 2.2. The AICP survey has been used historically to measure dispositional trust in autonomous systems (Merritt et al., 2019) and its inclusion as a predictor in our model demonstrates its validity. Similarly, the remaining surveys were captured as they were shown by Chung and Yang (Chung and Yang, 2024) to be significantly correlated with trust dynamics in how people will report trust during experiments (i.e., as Bayesian decision-makers, disbelievers, and oscillators). The inclusion of these surveys in our model corroborates their findings and indicates that dispositional attitudes play a role in a person’s feelings towards automation during the HAT task. Conversely to their results, though, the scores from the CVS and the Big Five Factors of Personality were not selected as predictors in the model. This may be because their study retrospectively identified important surveys, while the effort collected them prospectively, where the information they provided did not yield predictive utility beyond other features included in our models. Operator background measures related to demographic information included age and race as predictors in the overall model. No other demographic or cultural information was retained.

While operator background information including demographics and personality scores are indicative of a person’s history or values and thus provide insight into dispositional trust, these features may also aid in predicting how *dynamic* someone will be in trusting an autonomous system during a HAT task. In data collection, it was observed that some participants reported their trust as highly fluctuating between 0 and one throughout trials (e.g., participant four in Figure 9), whereas others reported their trust as consistently and steadily high or low (e.g., participant five in Figure 9). The findings from Chung and Yang show how a person’s personality may affect their moment-to-moment interactions with autonomous systems and how dynamic trust may be impacted by statically measured characteristics (Chung and Yang, 2024). This nuance further explains the operator background information in the context of our model. These features were used to capture inter-individual differences in both dispositional trust and trust dynamics.

Embedded measures were also specifically created to be dynamic indicators of trust. Features taken from actions the participants took are directly indicative of trust, containing the following features as trust predictors: time spent on the recommendation review screen, percent of satellites reviewed, percent of satellites ignored, percent of agreement with the autonomous system, percent of passive agreement with the autonomous system, average time between clicking the review button to agreeing/disagreeing with the autonomous system, and the percent of satellites re-reviewed. Each of these are per-epoch measures, calculated every 45 s to inform dynamic changes in trust. The participant agreement and time-to-classify measures are synonymous to embedded measures historically used in assessing trust and workload during road vehicle simulations (e.g., time to take over, braking behavior) (Petersen et al., 2019). Additionally, our calculation of embedded measures improves upon previous models (Hergeth et al., 2016; Kunze et al., 2019; Petersen et al., 2019; Walker et al., 2019), as they are taken on the same time scale as the dynamic trust reports.

Information relating to the participant’s secondary actions during the task involves their interaction with the scan guidance map. When performing the map task, participants opted to divert attention away from monitoring the autonomous system and towards a different objective. These measures capture trust by describing the participant’s strategy in division of attentional resources (e.g., if an operator trusts their autonomous system teammate more, they will spend more time in a passive role, completing other tasks). This type of feature as a predictor of trust cannot be decoupled from workload, as described in previous research (Yamani et al., 2020). Thus, while proven relevant to trust predictions via the feature down-selection process, it is important to note that measures associated with allocating attentional resources are not absolute indicators.

### Trust dynamics and dimensions

An additional key advantage of this research is the purposeful alteration of multiple dimensions of trust during data collection. Aspects of the task were designed to influence affective and cognitive trust dynamically through the explainability and reliability of the simulated autonomous system, respectively. Even though the trust sliders during the HAT task asked participants to rate their overall trust in the autonomous system and collected ratings as a single construct, the multi-dimensional components of the task aimed to yield a comprehensive measure of overall trust. While we did not explicitly measure affective and cognitive trust, our results indicate that our model is sensitive to multiple facets by which trust may be altered. Past studies have considered multidimensional trust in other contexts, such as between supervisors and subordinates, within teams, and between consumers and online retailers (Erdem and Ozen, 2003; Yang et al., 2009; Punyatoya, 2019). Existing research into affective and cognitive trust demonstrates the value in separately observing trust constructs, and our findings imply that trust in autonomous systems should be studied in a similar manner. Other HAT-specific research recognizes the multidimensional nature of trust, but, during data collection, only aim to alter cognitive trust (Shamim et al., 2023; Buchner et al., 2025). The results of our research attempt to account for emotional states (synonymous with affective trust) in addition to rational thoughts (synonymous with cognitive trust).

Another primary contribution of this research is the model’s ability to predict rapid changes in trust. This adds to the body of work aiming to fill the gap outlined by previous research, which states that trust-oriented experiments should sample trust more frequently (Kohn et al., 2021; Tenhundfeld et al., 2022; Rodriguez Rodriguez et al., 2023). Our results indicate that physiological data and embedded measures mapped to 45-s trust ratings provide a high temporal resolution in trust predictions. Though the model itself is static, it can infer rapid trust dynamics when provided with previously unseen data. This adds value to the field of research because it has applicability to possible real-time applications of trust measurement or adaptive autonomous systems, as highlighted in previous research (Feigh et al., 2012; Schwarz and Fuchs, 2018; Kintz et al., 2023). For operational settings real-time prediction is required for the models to yield improved performance. Dynamic trust modeling allows for the effects of discrete interactions with the autonomous system to be captured, rather than multiple minute-long, generalized observations, as has been done in previous studies (Hergeth et al., 2016; Kunze et al., 2019; Petersen et al., 2019; Walker et al., 2019). One limitation of this model, however, is that participants with extreme shifts in trust (as seen in participants four and six in Figure 9) between epochs are not modeled as well as those with more steady trust trends. This is likely due to the model being a cohort model, rather than twelve personalized models. Thus, there is a smoothing effect because the highly variable trust reports from some participants are combined with the highly steady trust reports from others. The advantages of cohort models, however, are that they are more generalizable to new populations or tasks, they are simpler to implement, and they help identify trends across a wide dataset. Previous studies that have used personalized models were limited in their ability to quickly generalize their models to a large group of people, even though they achieved high personalized predictive power (Guo and Yang, 2021). Additionally, any model using autoregression in place of OLS may be worse at capturing highly variable trust. Another advantage of the model in predicting trust dynamics is the wide range of trust values that are predicted. The HAT task sufficiently altered trust to capture both high and low ranges of trust reports, forming a rich dataset for use in modeling. Thus, we see that the trust dynamics of people who are consistently low or high trusters, such as participants three and five in Figure 9, are still captured, rather than skewed towards the middle.

### Model validity

In addition to accurate trust estimations and predictions, one of the key advantages of this model is the robust approach in feature down-selection using LASSO. Concerns regarding model stability and repeatability arise when building models with large sets of potential predictors. To mitigate instability and flaws in the model, we implemented relaxed LASSO and stability selection in which LASSO was run once on the original potential predictor set, then again on the new down-selected feature set in an effort to remove falsely selected variables (Meinshausen, 2007; Meinshausen and Bühlmann, 2010). This method has been implemented before internally (Buchner, 2022; Richardson et al., 2025) but is not typical in other models in the literature. Furthermore, an emphasis was placed on avoiding model overfitting when selecting the final model from the options generated during cross validation in the OLS step. The constraint of eliminating models where there were more predictors than 1/5 the number of observations helped to reduce model complexity, as a higher predictor-to-observation ratio could yield an overfit model (Austin and Steyerberg, 2015; Richardson et al., 2025). The additional constraint of only retaining models with a Q^2^ within a predetermined value (0.2) of the adjusted R^2^ further avoids overfitting, as large differences in these values indicate misaligned model fit and predictive capabilities. While the focus of this effort was not to determine the optimal features to make these predictions, across the 10 cross validation splits the model performance remained consistent, even when the same features were not always selected in the model fitting process.

Another novel aspect of this research is the validation approach used during model evaluation. Many existing models use an internal validation method where feature down-selection is conducted on the entire dataset. Our model was built with an external validation method in which feature down-selection was conducted on only a subset of the dataset and the model’s predictive capabilities are evaluated on the left-out data, making the model more robust and applicable to real-world scenarios. Internal validation methods involve assessing a model’s predictive performance using data from the same dataset that was used for training the model. This poses multiple risks to model validity, including potential for overfitting, limited generalizability to new datasets, and optimistic bias in the performance metrics (Collins et al., 2024). Conversely, external validation methods aim to address these limitations by using independent datasets in model creation and model evaluation.

To further strengthen the validity of the model, the single-item analog scales to which the model was fit were validated against the 12-item Jian Trust in Autonomous Systems Survey, which has been used historically in a large body of trust assessment research (Jian et al., 2000; Leary et al., 2024). Single-item sliders were implemented in the experiment to quickly and frequently capture subjective trust ratings without taking the participant’s attention away from the task at hand and were thus deemed minimally obtrusive. To ensure these sliders were appropriately capturing participants’ trust ratings, the Jian survey was administered at the end of each trial and later used to validate the single-item scales. Our validation achieved an Adjusted *R* ^*2*^ = 0.72 between the trust sliders and the Trust in Automation survey (Leary et al., 2024). This provided us with confidence that the simplified surveys were functioning as intended, and that the model was fit to valid measurements.

### Implications, limitations, and future work

If operator trust can be predicted accurately, including dynamically changing trust, in a non-disruptive manner, the relationships between humans and their autonomous system teammates can be better understood. Our model may enable the creation of systems that help to better calibrate trust, identify distrust or mistrust, and improve HAT performance. This could be done by having operators wear sensors during their workday such that the sensor system’s measures can be fed directly into the model, yielding a real-time, dynamic estimate of trust. The model also provides trust predictions for a HOTL scenario. Thus, it may be applied in environments where there exists spatial or temporal separation between the operator and the autonomous system, the operator has limited additional information, and the operator is in a supervisory position.

There are several limitations to this research. Our external validation models are not fully blind to every participant, as 1/6th of the cohort data was retained by removing one entire trial from each participant and each session before conducting feature down-selection and fitting the model. When the test and training data is split to remove all data for a given individual, the model’s predictive capabilities worsen due to high inter-individual differences among participants. Thus, the model is limited in its ability to predict completely unseen participants. However, this does not undermine the utility of the model in predicting rapid changes in trust. It becomes powerful when provided prior information such as operator background information and previous trials worth of physiological data. Future work could develop methods to achieve high performance when predicting unseen participants to make the model more easily generalizable without requiring additional data collection before use.

Our model building process also has a large feature space, many of which may be nearly co-linear. When developing the model-building process, LASSO was chosen as the predictor down-selection tool due to its strength in managing large predictor sets, performance in both variable selection and regularization, and ability to resolve multicollinearity (Buchner, 2022; An et al., 2024; Herawati, Nisa and Setiawan, no date). Relaxed LASSO, in particular, was used to address LASSO’s limitation of potential bias in parameter estimation when dealing with highly correlated variables (Meinshausen, 2007). Thus, all features and feature versions were provided to LASSO as potential predictors, and no additional efforts were taken to address the collinearity confound. However, the relaxed LASSO process was imperfect in removing all collinear predictors. This may mean that the model contains unstable coefficient estimates or unreliable predictions.

Future work should address the concern of sensor burden. In this work, we chose to include six psychophysiological sensors to provide a large suite of data streams and potential trust predictors, which is a strength of the model itself. It is, though, unrealistic that all these sensors may be worn at once in a real-world operational environment. These sensors are non-disruptive in that they do not directly detract attention from an operator’s task objectives, but they can often be obtrusive due to physical discomfort while wearing them for long periods of time (Buchner et al., 2025). While perceived obtrusiveness or discomfort varies based on the discipline, operational environment, and the operator’s personal preferences, any consistent and reliable use of physiological sensors requires operator acceptance (Eggemeier et al., 1991; Boggs et al., 2010). An ideal model of trust would achieve similar predictive and descriptive performance by utilizing fewer sensors. Future work may involve ablation studies to determine the minimum number of sensors needed to be worn to accomplish the same predictive strength. These efforts align with those of Buchner et al., who researched the utility of psychophysiological sensors in modeling trust, situation awareness, and mental workload. Our ablation studies would look at both psychophysiological and neurophysiological categories.

Our ongoing work is capturing both affective and cognitive dimensions of trust, through both independent sliders and surveys. We hypothesize that future models may be improved by estimating and predicting affective and cognitive trust as separate entities.

Finally, future work involves the use of trust models on live data streams in real time. The current model fits coefficients and computes trust values offline but still informs the capabilities of predicting trust unobtrusively. Real-time models may be able to utilize one to two bio-sensors, rather than all six, and still achieve similar results.

## Conclusion

This research contributes to our fundamental understanding of trust dynamics, as it shows how human operators may engage with an autonomous system in a rapid processing scenario. The combination of physiological signals with embedded measures and operator background can better non-disruptively predict trust. The operationally relevant and complex HOTL HAT task captured inherent trust dynamics. This is reflected in the model, which has high descriptive fit and predictive accuracy. As systems reach higher levels of autonomy, HOTL scenarios will become increasingly prevalent. Modeling supervisory control where the operator has limited context, limited information, and limited collaboration with the autonomous system has been previously under-studied. Our model bridges those gaps. It also models trust as dynamical in nature and captured multiple facets as components of overall trust. These qualities improve upon previous experiments and have the potential to increase transferability to other tasks.

## Data Availability

The raw data supporting the conclusions of this article will be made available by the authors, without undue reservation.
